# Supplementary material for: Patterns of Intron Gain and Loss in Fungi
Source: PLoS Biol. 2004 Nov 30;2(12):e422. doi: 10.1371/journal.pbio.0020422 (PMC532390; doi:10.1371/journal.pbio.0020422)
Supplement: Table S1 — Also available at http://genes.mit.edu/NielsenEtAl/. (4.3 MB ZIP). [file pbio.0020422.st001.zip › NielsenEtAl/html/928.html]

AN0200.1.NCU06311.1.MG08802.1.FG05007.1


```
 CLUSTAL W (1.82) Multiple Sequence Alignments - Introns Inserted


Sequence 1: AN0200.1	252 aa
Sequence 2: FG05007.1	246 aa
Sequence 3: NCU06311.1	307 aa
Sequence 4: MG08802.1	253 aa
Alignment Length: 311 aa
Number Identitical Residues: 77 aa
Alignment Score (without introns) 4576


MG08802.1 	----MAAKP-PTRLAILMADTPMPNTAAKYGDYGGLFTSLFT------------------
NCU06311.1	MSSPLTPPPRPIRLAILEADTPVPGAHARYSGYRGVFTHLFTRALASSSSPSSSSSSSSS
FG05007.1 	------MP--PLRLAILEADTPQPQTRDRYGGYTGVFTALLEAAAK--------------
AN0200.1  	------MPRTPLRIAVLECDTPPESSNAKYGGYVGVFKTLLYSSVR--------------
          	          * *:*:* .***   :  :*..* *:*. *:  :                

MG08802.1 	-----------TAAQPEPIESILSITKHDIVNHVGSYPSLDDVDAVLITGSK~HSAFADD
NCU06311.1	SSQDQDQDQKQQQGQQHPLSSYLTISAHDVVNNLSDYPSLSDIDAILITGSK~HSAYEND
FG05007.1 	---------PKK------LSELVSIKGYDVVNELHSYPVIEDIDAILITGSR~HTAFDND
AN0200.1  	---------ELYKQEEVDPSSILEISRFDVVTAQN-YPDLANVDAVLLTGSK1HNSFEDH
          	              .    .. : *. .*:*.    ** : ::**:*:***: *.:: :.

MG08802.1 	AWIMALVDYTRRALLETNGR----------------------VKVVGICFGHQIVARALG
NCU06311.1	PWIVKLTEFVKKVLTEQEGDGDGDGDGDSDDGSIIKGGKKKIIKVIGVCFGHQIIGRALG
FG05007.1 	PWILKLVEYTKKAIAS------------------------NRVRVVGVCFGHQIVGRAEG
AN0200.1  	PWILKLVEFTKKAIEH------------------------PRVKLLGICFGHQIIGRALG
          	.**: *.::.::.:                            ::::*:******:.** *

MG08802.1 	GKVVVNEKGWEVAVTEVELTERAREFFG-LETL0RIHQMHRDIVPELPKTDPPTMLLASN
NCU06311.1	QVVERNEKGWEVSVTPVGLTDVGRRLFEGMEEL0KIQQMHRDHVVGVPEG---AQLLAST
FG05007.1 	AKCGRSNNGWEVAVTEVDLTAKGKEVFG-LDKM0RIHQMHRDIVDAFPKN---SIPLGSN
AN0200.1  	VEVGRNSAGWEIAVCDVDLTEKGKELFG-VETL0KIQQMHRDIVFAYPDG---VTPLGSS
          	     .. ***::*  * **  .:..*  :: : :*:***** *   *.       *.*.

MG08802.1 	NVCQVQGMLWPGRCVTVQGHPEFTEEVVREVLETRHDAGIHPDDLYNSGISRVANQHDGI
NCU06311.1	DVCENQGFVIPGRVLTVQGHPEFTTDIMEELLELRKGTGLFNEELFGSGMERNK-VDDGV
FG05007.1 	NICEVQGFYSPGKYITVQGHPEFNEEIISEILFNRHTVGIFTDEVYNDAMKRAPLPHDGV
AN0200.1  	PRCEVQGMYKAGKFITVQGHPEFKEDIVSEVVNLRAAAGVFDKGQAEDALERAGKPHDGI
          	  *: **:  .*: :********. ::: *::  *  .*:. .    ..:.*    .**:

MG08802.1 	AIAKGFLKFLRL-
NCU06311.1	FIAQAFYKFLLQD
FG05007.1 	AIAKAFLKFWREG
AN0200.1  	AIGVAFLKFLLE-
          	 *. .* **
```
